# Levodopa improves handwriting and instrumental tasks in previously treated patients with Parkinson’s disease

**DOI:** 10.1007/s00702-020-02246-3

**Published:** 2020-08-19

**Authors:** Thomas Müller, Ali Harati

**Affiliations:** 1 Department of Neurology, St. Joseph Hospital Berlin-Weissensee, Gartenstr. 1, 13088 Berlin, Germany; 2 Private Practice for Neurosurgery, MVZ PAN Institute GmbH within the Department of Neurosurgery, Pan Klinik Am Neumarkt, Zeppelinstr 1 Neumarkt-Galerie, 50667 Cologne, Germany

**Keywords:** Levodopa, Parkinson’s disease, Motor behavior, Handwriting

## Abstract

Motor symptoms in patients with Parkinson’s disease may be determined with instrumental tests and rating procedures. Their outcomes reflect the functioning and the impairment of the individual patient when patients are tested off and on dopamine substituting drugs. Objectives were to investigate whether the execution speed of a handwriting task, instrumentally assessed fine motor behavior, and rating scores improve after soluble levodopa application. 38 right-handed patients were taken off their regular drug therapy for at least 12 h before scoring, handwriting, and performance of instrumental devices before and 1 h after 100 mg levodopa intake. The outcomes of all performed procedures improved. The easy-to-perform handwriting task and the instrumental tests demand for fast and precise execution of movement sequences with considerable cognitive load in the domains' attention and concentration. These investigations may serve as additional tools for the testing of the dopaminergic response.

## Introduction

**Fig. 1:**
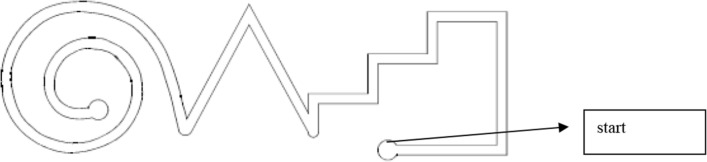
Graph from the line tracing task ( modified from https://psydok.psycharchives.de/jspui/bitstream/20.500.11780/1018/1/Mls.pdf, page 7)

Neurological examination of patients with Parkinson’s disease (PD) includes several procedures that determine the impaired function of motor behavior. One is the performance of clinical scoring of motor symptoms with validated rating scales, like the Unified Parkinson’s disease Rating Scale (UPDRS) (Fahn et al. 1987). However, this evaluation of motor impairment in PD may vary between different examiners and may relatively be insensitive to subtle modifications. The subjective impression of the patient by the rating neurologist may additionally impact outcomes (Goetz et al. 2008). Accordingly, quantitative standardized instrumental procedures for objective assessment of motor behavior have been developed over the years (Lee et al. 2016; Lopane et al. 2018). The employed technical methods aim to assess motor symptoms, particularly tremor and slowness of movement, which is considerably biased by rigidity (Goetz et al. 2008; van Uem et al. 2016; Li et al. 2018; Lopane et al. 2018; Bertoli et al. 2019). Particularly tremor reduces the precision of aimed movements, whereas bradykinesia and rigidity support delay or failure to initiate a willed movement and slowness of an ongoing motion sequence (Lalonde and Botez-Marquard 1997; Haaland et al. 2004). This triad of main cardinal motor symptoms in PD patients influences instrumentally assessed execution of complex movement series (Müller et al. 2017). They are sensitive to dopamine substitution in PD patients, as it has been shown with the peg insertion paradigm, which resembles the rather popular, simpler purdue pegboard task (Müller et al. 2005). As an example, instrumental assessment of conduction of complex movement sequences showed good test–retest reliability and correlated to the nigrostriatal dopamine deficit in PD (Pal et al. 2001; Müller et al. 2002). The outcomes of employed instrumental methods reflected altered motor behavior not only in PD, but also in multiple sclerosis or Huntington’s disease (as examples: (Ringendahl 2002; Andrich et al. 2007)). Execution of more complex movement series involves higher cortical functions and dopamine sensitive mesolimbic structures. The necessary high cognitive load also demands attention and motivation (Nieoullon and Coquerel 2003; Bidet-Ildei et al. 2011; Trujillo et al. 2019). Similar cognitive efforts with a hypothetical involvement of resembling brain structures are necessary when one writes a sentence (Gangadhar et al. 2009). It is well known that not only micrographia, which is abnormal small, cramped handwriting or the progression to progressively smaller handwriting, but also bradykinesia and rigidity influence the handwriting procedure in PD patients (Lalonde et al. 1997; Nieoullon et al. 2003; Lange et al. 2006; Broeder et al. 2014; Nackaerts et al. 2017b). To a certain extent, a resembling instrumental task is the performance of a line tracing task, which asks the individual to follow a given path (Fig. 1). Measurements concern the execution velocity, the number, and duration of contacts to the path (Müller et al. 2005). The execution of instrumental tests in combination with a rating procedure may provide additional useful information on the functioning and the impairment of the individual PD patient in particular when patients are tested off and on medication to evaluate the dopaminergic response (Gelb et al. 1999). The objective was to determine whether outcomes of aforementioned instrumental tests and a standardized writing task may reflect the dopaminergic response similar to the improvement of clinical rating scale scores following levodopa/benserazide application (Müller et al. 2002, 2003, 2017).

## Methods

### Subjects

Participants were 38 treated, right-handed, idiopathic PD patients (see Table 1) without unpredictable motor fluctuations. Individuals with medical conditions, which may affect the outcomes of the performed instrumental tests, were excluded.

**Table 1:** Characteristics of participating PD patients

Age	61.15 ± 18.54 years
Sex	20 men; 18 women
Duration of PD	4.26 ± 4.86 years
Age of onset	53.87 ± 13.01 years
LEED	386.45 ± 261.70 mg
HYS	2.01 ± 0.85
UPDRS I	2.63 ± 2.06
UPDRS II	9.84 ± 6.34
UPDRS IV	1.09 ± 0.12
UPDRS III right	10.37 ± 5.65
UPDRS III left	9.63 ± 5.06
PD dominance	8 equal, 14 left, 16 right

*HYS* Hoehn and Yahr Scale, *LEED* L-dopa equivalent daily dose, *PD dominance* most affected side by the disease process (equal = equal on both sides, left = left sided, right = right sided), *UPDRS I* unified Parkinson’s disease rating scale mental behavior, *UPDRS II* unified Parkinson’s disease rating scale activities of daily living, *UPDRS III* unified Parkinson’s disease rating scale motor examination, *UPDRS IV* unified Parkinson’s disease rating scale motor complications, *UPDRS III right/left* items 20, 21, 22, 23, 24, 25, 26 (right/left) of unified Parkinson’s disease rating scale

### Design

Hospitalized patients were taken off their regular PD drug therapy for at least 12 h before the rating and execution of devices. First the rating (A.H.), second the handwriting test (A.H.), and third the assessments with the devices [standardized sequence: peg insertion [first], line tracing [second], were performed (technicians)]. Then, the patients received one tablet of 100 mg levodopa (L-dopa)/25 mg benserazide, dissolved in 100 ml water (Madopar LT^®^). One hour later, UPDRS III rating and the instrumental tests in the same sequence were again executed. To minimize learning and training effects, all PD patients were allowed to practice for 1 min on the day before with all instrumental tests.

### Rating

Motor symptoms were scored with the part motor examination (III) of the UPDRS (Fahn et al. 1987).

### Writing test

The participants were asked to sit down in a comfortable position and then to write the same sentence, consisting of 5 words with 24 characters (“Bochum ist eine schöne Stadt”), for three times on white paper without lines. Initially, subjects were asked to write this sentence two times. The patients were instructed to write this sentence with a comfortably speed like in the daily routine and not write this sentence as fast as possible. Then, the assessment of the needed interval for the handwriting task was performed. A stopwatch with 100 ms accuracy was employed.

### Instrumental tests

#### Peg insertion

Subjects were instructed to transfer 25 pegs (diameter 2.5 mm, length 5 cm) from a rack into one of 25 holes (diameter 2.8 mm) in a computer-based contact board individually and as quickly as possible. The distance between rack and appropriate holes was exactly 32 cm. The board was positioned in the middle and the task was carried out with the right hand only. When transferring each peg from rack to hole, elbows were allowed to be in contact with the table. The interval between the inserting of the first and the last pin was measured by a computer with an accuracy of 100 ms (Müller et al. 2005). In case of dropping of one pin, the instruction was to repeat this task.

#### Line tracing

The patient was asked to follow a grooved path with a stylus as exact and fast as possible from the right to the left side with the right hand one time only. The total test duration, the number of contacts, and the length of contacts to the panel interfacing with a computer, which recorded all these parameters, were assessed (Müller et al. 2005). Intervals were determined with 100 ms accuracy.

### Statistics

A non-parametric data distribution was shown according to the Kolmogorov–Smirnov test outcomes mainly. Therefore, non-parametric tests were only employed for this exploratory analysis. The Wilcoxon matched-pairs test was used for comparisons and Spearman rank correlation for correlation analysis. Suitable items of UPDRS part III were selected for calculation of subscores, i.e., the UPDRS III score of the right arm (items: 20, 21, 22, 23, 24, 25 [right arm]) only. The differences between the outcomes of the two assessment moments were calculated according to the formula: outcome before L-dopa/benserazide intake—outcome 1 h after L-dopa/benserazide = difference. Four different assessment tools, the peg insertion task, the line tracing paradigm, the writing test, and the UPDRS III rating were performed. Therefore, the significance level was corrected to p < 0.0125 for both comparisons and the correlation analysis. P-values between 0.0125 and 0.05 were discussed as a significant trend.

### Ethics

The study was approved by the local institutional ethics committee of the Ruhr University of Bochum. The study was performed in the Department of Neurology, St Joseph Hospital (Head at that time: Professor Dr. H. Przuntek). The investigation represented a non-interventional study, i.e., the rules imposed for this observational plan did not interfere with the physician’s common therapy. Patient’s written informed consent regarding the forwarding and storing of medical data according to GDPR laws was obtained.

## Results

### Comparisons

**Fig. 2:**
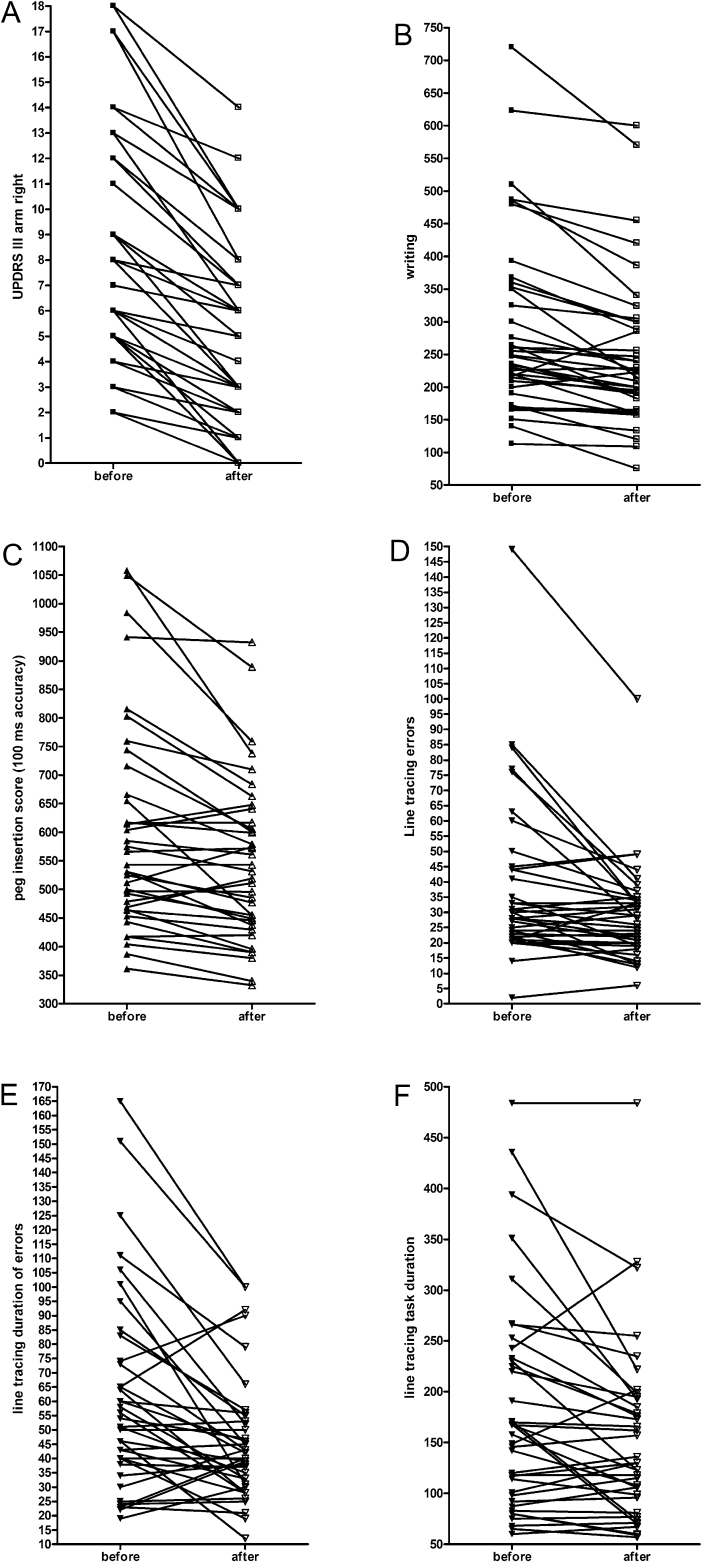
Decline of the UPDRS part III arm scores (**a**), writing interval (**b**), peg insertion result (**c**), number of line tracing errors (**d**), line tracing duration of errors (**e**), interval of line tracing (**f**) before and 1 h after intake of 100-mg L-dopa/25-mg benserazide. *UPDRS III* motor examination (III) of the Unified Parkinson’s disease rating scale, *y*-axis of **b**, **c**, **e**, **f** describes data with 100-ms accuracy

As to be expected, there was a decrease and thus an amelioration of the UPDRS part III total score (before L-dopa/benserazide intake [before]: 27.92 ± 13.90 [mean ± SD]; 1 h after L-dopa intake [after]: 18.95 ± 12.37, *p* =  < 0.0001). Accordingly, the UPDRS part III right arm subscore also went down and improved (Fig. 2a, *p* =  < 0.0001). Table 2 shows the outcomes of further UPDRS III subscores. The interval for the writing task performance declined (Table 2, Fig. 2b). The execution of the instrumental tests also improved after L-dopa/benserazide intake (Table 2; peg insertion outcome right side: Fig. 2c; line tracing errors: Fig. 2d; line tracing duration of errors: Fig. 2e; line tracing total interval: Fig. 2f).

**Table 2:** Comparisons of data before and after L-dopa intake

	Before	After	Difference	*p*
UPDRS III	27.42 ± 13.90	18.95 ± 12.37	8.97 ± 6.64	< 0.0001
UPDRS III right arm	8.24 ± 4.62	5 ± 3.57	3.24 ± 2.13	< 0.0001
UPDRS III right arm rigidity	1.40 ± 0.89	0.95 ± 0.90	0.45 ± 0.64	0.0005
UPDRS III right arm bradykinesia	4.63 ± 2.78	2.95 ± 2.44	1.68 ± 1.70	< 0.0001
UPDRS III right arm tremor	1.95 ± 1.87	1.03 ± 1.26	0.92 ± 1.04	< 0.0001
Writing	290.55 ± 134.39	249.16 ± 113.42	41.39 ± 44.99	< 0.0001
Peg insertion	596.74 ± 181.16	543.37 ± 140.13	53.37 ± 78.37	0.0002
Line tracing errors	38.24 ± 26.64	28.95 ± 15.42	9.29 ± 16.19	0.0015
Line tracing duration of errors	61.55 ± 34.63	45.87 ± 21.39	15.68 ± 24.22	0.0011
Line tracing total interval	181.58 ± 104.35	153.66 ± 86.56	27.92 ± 56.32	0.0055

All data are given as mean ± standard deviation

*After* 1 h after intake of 100 mg L-dopa/25 mg benserazide, *before* before intake of 100-mg L-dopa/25-mg benserazide, *p* *p* value; data of instrumental tests and writing are given with 100-ms accuracy except line tracing number of errors; UPDRS III right arm, items 20, 21, 22, 23, 24, 25 (right arm) of Unified Parkinson’s Disease Rating Scale; UPDRS III right arm rigidity; item 22 (right arm) of Unified Parkinson’s Disease Rating Scale; UPDRS III right arm bradykinesia; items 23, 24, 25 (right arm) of Unified Parkinson’s Disease Rating Scale; UPDRS III right arm tremor; items 20, 21 (right arm) of Unified Parkinson’s Disease Rating Scale

### Correlations analysis

The results are shown in Table 3. Only significant outcomes and associations with a significant trend are reported.

**Table 3:** Correlation analysis of parameters before and one hour after L-dopa intake and the corresponding computed differences

Line	Variable 1	Variable 2	*R*	*p*
Before				
1	Writing	Peg insertion right	0,44	0,0056
2	UPDRS III right arm	Peg insertion right	0,48	0,0021
3	UPDRS III right arm	Line tracing errors	0,37	0,0213
4	UPDRS III right arm	Line tracing duration of errors	0,66	< 0.0001
5	UPDRS III right arm	Line tracing total interval	0,39	0,0143
6	Peg insertion right	Line tracing errors	0,32	0,0477
7	UPDRS III right arm bradykinesia	Peg insertion right	0,48	0,0024
8	UPDRS III right arm bradykinesia	Line tracing duration of errors	0,62	< 0.0001
9	UPDRS III right arm bradykinesia	Line tracing total interval	0,42	0,0086
10	UPDRS III right arm rigidity	Line tracing errors	0,33	0,0454
11	UPDRS III right arm rigidity	Line tracing total interval	0,52	0,0007
12	Peg insertion right	Line tracing duration of errors	0,55	0,0004
13	Line tracing errors	Line tracing duration of errors	0,45	0,0042
14	Line tracing errors	Line tracing total interval	0,65	< 0.0001
15	Line tracing duration of errors	Line tracing total interval	0,58	0,0001
After				
16	Writing	Line tracing duration of errors	0,32	0,0487
17	UPDRS right arm	Peg insertion right	0,47	0,0031
18	UPDRS right arm	Line tracing duration of errors	0,37	0,0233
19	Peg insertion right	Line tracing duration of errors	0,60	< 0.0001
20	Line tracing errors	Line tracing total interval	0,56	0.0003
21	UPDRS III right arm bradykinesia	Peg insertion right	0,51	0,0012
22	UPDRS III right arm tremor	Line tracing duration of errors	0,46	0,0077
Difference				
23	Peg insertion right	Line tracing errors	0.36	0.028
24	Line tracing errors	Line tracing total interval	0.49	0.0018
25	Line tracing errors	Line tracing duration of errors	0.37	0.024
26	Line tracing duration of errors	Line tracing total interval	0.54	0.0005

*after* 1 h after application of soluble 100 mg L-dopa/25 mg benserazide, *before* before intake of soluble 100 mg L-dopa/25 mg benserazide, *difference* computed differences between the outcomes of the two assessment moments according to the formula (outcome before – outcome after = difference); *R* correlation coefficient, *p* *p* value, *UPDRS arm* partial arm score motor examination (III) of the Unified Parkinson’s Disease Rating Scale

### Peg insertion

The outcomes were associated with the UPDRS III right arm score, the writing task, and the line tracing duration of errors before L-dopa intake (Table 3; lines 1, 2, 7, 12). These associations were also found following L-dopa intake with the exception of the correlation between the writing outcomes and peg insertion (Table 3; lines 17, 19, 21).

### Line tracing

There were close relationships between each of the determined parameters of the line tracing task before L-dopa intake (Table 3; lines 13–15). The number of these significant relations between all parameters of the line tracing task went down after L-dopa intake (Table 3; line 20). Nearly all the computed differences of each line tracing parameter correlated with each other, line tracing errors, and line tracing duration of errors showed a significant trend only (Table 3; lines 24–26). There was a significant trend for a correlation between the line tracing duration of error results and the writing results after L-dopa intake only (Table 3, line 16).

### UPDRS part III right arm score and further UPDRS III subscores

The rating scores correlated with the peg insertion results and with the line tracing duration of errors at baseline (Table 3; lines 2, 4, 8), and bradykinesia and rigidity were closely associated with the line tracing duration of interval (Table 3; lines 9, 11). 1 h after L-dopa intake, a correlation appeared between line tracing duration of errors and the tremor score (Table 3; line 22). As to be expected, there were close relationships between the various UPDRS III subscores (results not shown).

## Discussion

Particularly, bradykinesia and rigidity show execution of movement series. These two motor symptoms respond to dopamine substitution quite well. However, both the handwriting procedure and the instrumental test performances do not only depend on velocity. The initiation and conduction of the necessary precise and aimed movement sequences considerably demand cognitive load in the domains' attention and concentration (Lalonde et al. 1997; Espay et al. 2009; Cools et al. 2019). Similar abilities are also needed for the execution of the employed instrumental tasks. Accordingly, we show that all outcomes improved following standardized application of L-dopa/benserazide. We suggest this easy-to-perform handwriting test as an additional tool to supplement the clinical examination of a PD patient when the L-dopa response is tested, i.e., as an essential criterion of the diagnosis of PD (Gelb et al. 1999; Navailles et al. 2014; Trujillo et al. 2019). One must acknowledge that both handwriting and the employed apparatus methods only focus on fine motor behavior of the right hand, which limits their suitability for the clinical use in the case of PD patients with onset of predominant or only left-sided motor symptoms. Prior investigations on handwriting and associated micrographia with digital systems employed tablets with electronic pens. These trials mostly focussed on script height to distinguish PD patients from healthy controls, whereas the speed of task execution sometimes played a minor role in the data analysis (Popovic et al. 2008; Ponsen et al. 2008; Bidet-Ildei et al. 2011; Rosenblum et al. 2013; Cascarano et al. 2019; Zham et al. 2019). The collected data of the handwriting scenario in our present trial mainly assess writing velocity only. We stress that handwriting also asks for readability in the specific investigational situation, which is considerably influenced by precise trajectories with their considerable impact on size of letters, speed, and fluency of writing (Nackaerts et al. 2017a, b). Coordinated performance and accurated performance of motion sequences are characteristics, which are also important for the execution of the peg insertion test. This task demands to insert a peg into a hole with an aimed movement pattern (Müller et al. 2002). The instrumental determination of number of errors and duration of errors in the line tracing task in particular mirrors the functional capacity for execution of courses of aimed and thus precise movement series (Müller et al. 2000; Nieoullon et al. 2003). Accordingly, peg insertion outcomes showed close correlations to the duration of errors of the line tracing task mainly. Unlike the handwriting task, both peg insertion and line tracing are performed from the right to the left side. Inserting of pegs with its repeat performance and hand writing are influenced by training, which is different from the single carrying out of the line tracing task (Lalonde et al. 1997). This effect may hypothetically explain the missing association between handwriting and line tracing. Both instrumental test results were closely related to the UPDRS part III rating scores of motor behavior, which mainly reflect tremor, bradykinesia, and rigidity. One may assume that they also reflect the speed and precision of movement execution in a more indirect fashion. The aforementioned associations appeared before L-dopa intake. Following L-dopa intake, correlations coefficients of these associations were lower. The computed differences only showed relevant significant correlations within the various parameters of the line tracing task. Reasons for these missing relations may be that we assessed the L-dopa response one time only. We did not consider the different onset and time to maximum response to L-dopa. This effect is influenced by various components, such as duodenal absorption, gastrointestinal motility, and amino acid transport system activity. Repeated evaluations, i.e., every 30 min up to 2 h after L-dopa intake in view of the L-dopa plasma half-life, would have improved the quality of the present investigation (Müller et al. 2003). A further limitation is that we did not perform this trial in healthy controls to demonstrate or to exclude the learning effects and in previously untreated PD patients. As we included only treated PD patients, we cannot definitely exclude an effect of a long-duration response of the concomitantly performed PD drug therapy despite the 12 h lasting washout period. Therefore, we suggest further research in previously untreated PD patients. These future investigations will aim to confirm whether line tracing duration of error outcomes may serve as a specific marker for tremor and bradykinesia. Such a study will also focus on further putative relationships between scores of rigidity, respectively, bradykinesia and the various line tracing parameters.

In conclusion, we show that the performance of a simple handwriting paradigm in combination with resembling instrumental tasks may reflect an improvement of the velocity of movement execution following the administration of soluble L-dopa/benserazide in previously treated PD patients.
